# PVC containing silver nanoparticles with antimicrobial properties effective against SARS-CoV-2

**DOI:** 10.3389/fchem.2023.1083399

**Published:** 2023-03-13

**Authors:** Daniel J. da Silva, Guilherme B. Gramcianinov, Pamela Z. Jorge, Vanessa B. Malaquias, Augusto A. Mori, Mário H. Hirata, Sergio A. M. Lopes, Luciano A. Bueno, Mathilde Champeau, Danilo J. Carastan

**Affiliations:** ^1^ Center for Engineering, Modeling, and Applied Social Sciences (CECS), Federal University of ABC (UFABC), Santo André, SP, Brazil; ^2^ Department of Clinical and Toxicological Analysis, Faculty of Pharmaceutical Sciences, University of São Paulo, São Paulo, SP, Brazil; ^3^ BRGoods Indústria e Comércio de Produtos Hospitalares, Indaiatuba, SP, Brazil

**Keywords:** SARS-CoV-2, poly(vinyl chloride), COVID-19, silver, nanocomposites

## Abstract

Poly (vinyl chloride) (PVC) is commonly used to manufacture biomedical devices and hospital components, but it does not present antimicrobial activity enough to prevent biofouling. With the emergence of new microorganisms and viruses, such as Severe Acute Respiratory Syndrome Coronavirus 2 (SARS-CoV-2) that was responsible for the global pandemic caused by Coronavirus Disease 2019 (COVID-19), it is evident the importance of the development of self-disinfectant PVC for hospital environments and medical clinics where infected people remain for a long time. In this contribution, PVC nanocomposites with silver nanoparticles (AgNPs) were prepared in the molten state. AgNPs are well-known as antimicrobial agents suitable for designing antimicrobial polymer nanocomposites. Adding 0.1 to 0.5 wt% AgNPs significantly reduced Young’s modulus and ultimate tensile strength of PVC due to the emergence of microstructural defects in the PVC/AgNP nanocomposites, but the impact strength did not change significantly. Furthermore, nanocomposites have a higher yellowness index (YI) and lower optical bandgap values than PVC. The PVC/AgNP nanocomposites present virucidal activity against SARS-CoV-2 (B.1.1.28 strain) within 48 h when the AgNP content is at least 0.3 wt%, suitable for manufacturing furniture and hospital equipment with self-disinfectant capacity to avoid secondary routes of COVID-19 contagion.

## 1 Introduction

Poly (vinyl chloride) (PVC) has been used in the medical field for over three decades, widely applied in medical applications today because it is impervious to germs, easily cleaned, and allows sterilization and disposable applications that reduce healthcare infections (Zhao and Courtney, 2009; Wypych, 2016). In addition to applications in the biomedical sector, PVC has been used in bottles, cables, domestic appliances, pipes, food contact films, among others (Schiller, 2015). The reasons for the extensive technological applications of PVC, including healthcare and packaging industry, is also due to the unique combination of properties, just to name a few, flexibility, transparency, chemical stability, biocompatibility and resilience, ease of processing, accessibility (cost and marketing), and recyclability.

Stabilizers and processing additives are indispensable to prevent the degradation of PVC during its thermal processing to guarantee the confection of biomedical devices and structural parts with suitable mechanical performance. Pristine PVC can present bacteriostatic activity against some bacteria (Zhao and Courtney, 2009; Schiller, 2015). However, stabilized and plasticized PVC does not present enough antimicrobial properties to impede biofilm formation since phthalate ester plasticizers, crazing, and other surface defects from UV exposure make additive PVC more susceptible to biofouling (Mark, 2004). Then, it is necessary to apply biocide additives in PVC formulations because it is vulnerable to biofilm formation due to the growth of multilayer bacterial colonies covered by an extracellular matrix composed mainly of polysaccharides (Ferreira et al., 2015).

Although controversies and doubts about the effects occasioned by stabilizers and processing additives on human health along with short and long-term exposure times, PVC has been considered an excellent material with biocompatibility, chemical stability, and sterilization resistance combined with economic advances that make this polymer one of the main materials used in the manufacture of products of extreme importance in medicine, such as flexible blood containers, urine ostomy bags, flexible tubes, inhalation masks, oxygen masks, and personal protective equipment (Zhong et al., 2013; Lewandowski and Skórczewska, 2022).

Since 2019, the Severe Acute Respiratory Syndrome Coronavirus 2 (SARS-CoV-2) and its variants have caused a system collapse and brought about health systems and health crises in several countries (Tao et al., 2021). SARS-CoV-2 and variants are highly contagious viruses transmitted between humans mainly through respiratory droplets *via* aerosol (Howard et al., 2020; Li et al., 2020). It has been alarmed that SARS-CoV-2 can maintain its potential for contagion even after 24 h on the surface of polymeric materials (van Doremalen et al., 2020). Therefore, PVC with self-disinfecting capacity is relevant to produce structural components and products, such as handrails and wall guards, to prevent critical epidemiological issues in hospital environments and medical clinics (Balagna et al., 2020; Hasan et al., 2020).

The main procedures to confer auto-disinfectant properties to PVC are surface modification and mixing inorganic materials with intrinsic bactericidal and fungicidal properties (Behboudi et al., 2018). The blending with cationic polymers and functionalization with cationic groups (cationization) are other viable technological approaches to add bactericidal properties to PVC (Palencia et al., 2019). The incorporation of antimicrobial agents in the polymer matrix may bring some advantages over other methods, such as the possibility of using conventional polymer processing equipment (extruders, injectors, among others) and longer time extension of the antimicrobial activity over time. However, the development of composites by this route generally requires high amounts of antimicrobial agents to achieve a bactericidal effect and not just a bacteriostatic activity. The development of polymer nanocomposites by using antimicrobial agents in the nanoscale can be an alternative way to avoid this problem. In concern of COVID-19 spreading, such technological approaches to develop self-sanitizing PVC are suitable to avoid the secondary routes of COVID-19 contagion, mainly in hospitals and healthcare clinics that involve touching a contaminated surface and then contamination with dirty hands as extensively reviewed in the literature (Marquès and Domingo, 2021; Correia et al., 2022).

Silver (Ag), copper (Cu), TiO_2_, ZnO, Cu_2_O, and CuO are the main inorganic antimicrobial agents applied for the development of antimicrobial materials (Sedighi et al., 2014; Gold et al., 2018; Vodnar et al., 2020). They act mainly by generating reactive oxygen species (ROS) and releasing metal ions that cause irreversible damage to biological components present in the viral structure and bacterial and fungal cells (Tan et al., 2019; Zhou et al., 2020). Several authors have shown the outstanding antimicrobial activity of silver nanoparticles (AgNPs) or silver-based nanoparticles over the other antimicrobial agents in polymeric nanocomposites (Pongnop et al., 2011; Narayanan and Han, 2017; Oliani et al., 2017; Shah et al., 2018; Kraśniewska et al., 2020; Morais et al., 2020; Rahimi et al., 2020), including against SARS-CoV-2 (Assis et al., 2021). A few studies have shown the auto-disinfectant ability of PVC/AgNP nanocomposites (Zampino et al., 2011; Azlin-Hasim et al., 2016; El-Sayed et al., 2016; Braga et al., 2018), but their antiviral capability against SARS-CoV-2 has not been investigated. Furthermore, it is important to mention that most of these works were not carried out by mixing PVC and AgNP in the molten state (Azlin-Hasim et al., 2016; El-Sayed et al., 2016; Braga et al., 2018). Generally, PVC/AgNP nanocomposites are prepared by solvent methods (typically casting and AgNP synthesis in the presence of dissolved PVC) that are not suitable for obtaining large products on an industrial scale. This contribution aims to fill this gap in the literature. Moreover, we evaluated the thermal stability, and mechanical properties of the PVC/AgNP nanocomposites.

## 2 Materials and methods

### 2.1 Materials

A rigid PVC compound in the form of pellets was supplied by Karina Plásticos (Brazil). AgNP liquid suspension (NpAg-925ETG) was purchased from TechNano Solutions (TNS, Brazil). HNO_3_ (65%), AgNO_3_ (99%), KSCN (>99%), Zn(NO_3_)_2_∙6H_2_O (96%–103%), Cu(NO_3_)_2_∙3H_2_O (98%–102%), and Fe(NO_3_)_3_∙9H_2_O (≥99.95%) were purchased from Synth (Brazil). All reagents were used as purchased without prior purification.

### 2.2 Methods

#### 2.2.1 Preparation of the PVC/AgNP nanocomposites

The PVC and PVC/AgNP nanocomposites were prepared through melt processing in an internal mixer (Model 50EHT 3Z, Brabender GmBh & Co. KG, Germany) at 160°C and a rotor speed of 60 rpm. First, PVC (50 g) was plasticized for 2 min, and then the AgNP suspension was added (0.5, 1, and 2 mL). The PVC samples were mixed for 8–10 min, using a fill factor of 80%. The nanocomposites were named PVC/*X*AgNP, where *X* corresponds to the AgNP content (0, 0.1, 0.3, and 0.5 wt%). The AgNP concentrations were estimated from the metal content measurements using Inductively Coupled Plasma Atomic Emission Optical Spectroscopy (ICP-OES).

Samples for impact testing were injected at 180 °C (test specimen dimensions according to ASTM D256 in a microinjection molder (Model 12cc, XPlore Instruments BV, The Netherlands), with mold temperature at 40 °C and 9 bar of pressure. The tensile samples were pressed in a hydraulic press (model SL 11, Solab Científica, Brazil) using a mold at 190°C, a residence time of 3 min, followed by 6 tons of pressure for 5 min. Then, the films (thickness = 1 mm) were wedge-cut in the specimen shapes following ASTM D1708.

### 2.3 Characterization

#### 2.3.1 AgNP suspension

##### 2.3.1.1 Dynamic light scattering (DLS)

The AgNP hydrodynamic diameter was characterized by dynamic light scattering (DLS), with a stable 90° scattering angle, using a Zetasizer Nano-ZS (Malvern Panalytical Ltd., Malvern, UK). The AgNP liquid suspension (50 μL) was diluted in distilled water (2 mL) before the DLS measurements.

##### 2.3.1.2 Zeta potential (ζ)

The Zeta potential (ζ) was calculated with the Smoluchowski model using electrophoretic mobility measurements of the nanoparticles obtained by Zetasizer Nano-ZS (Malvern Instruments, UK). The reading time to measure the Zeta potential data was 10 s, and the measurements were performed in duplicate.

##### 2.3.1.3 Energy-dispersive X-ray spectroscopy (EDS)

EDS spectra were obtained using a JEOL compact scanning electron microscope (JSM-6010LA) using the secondary electron detector (SEI). The AgNP suspension (∼20 μL) was previously deposited on carbon tape and then dried on a heating plate (300°C) in the ambient atmosphere.

##### 2.3.1.4 Fourier-transform infrared absorption spectroscopy (FTIR)

Fourier-transform infrared absorption spectroscopy (FTIR) measurements were performed on a Thermo IS5 Nicolet spectrometer, using an attenuated total reflectance (ATR) accessory (ZnSe crystal). Spectral data acquisition was conducted in the range of 600–4,000 cm^-1^, using 32 scans and a spectral resolution of 2 cm^-1^. Before FTIR measurements, the AgNP suspension was previously deposited (2 drops) on KBr pellets and dried at 100 °C for 30 min in a vacuum oven (Vacuoterm).

##### 2.3.1.5 Ultraviolet-visible absorption spectroscopy (UV-Vis spectroscopy)

UV-Vis spectroscopy measurements were performed using a UV-Vis spectrophotometer (Varian Cary, Model 50). The AgNP suspension was diluted in distilled water, and then the UV-Vis spectrum was collected.

##### 2.3.1.6 Inductively Coupled Plasma Atomic Emission Optical Spectroscopy (ICP-OES)

The silver, zinc, and copper content in the AgNP suspension was quantitatively estimated by ICP-OES analysis. The measurements were performed in an equipment ICP-OES Axial View, model 710 Series (Varian). The instrumental conditions are detailed in (Supplementary Table S1). The calibration curve was prepared from AgNO_3_, Cu(NO_3_)_2_, and Zn(NO_3_)_2_ aqueous solutions (HNO_3_-3%).

#### 2.3.2 PVC/AgNP nanocomposites

##### 2.3.2.1 Scanning electron microscopy (SEM)

The samples with PVC were coated with a 20 nm thick gold layer, using Leica EM ACE 200 Sputter Coater (Leica Microsystems, Wetzlar, Germany). Micrographs were taken in a microscope FEI Quanta 250 (Thermo Fisher Scientific, Hillsboro, Oregon, United States), using an accelerating voltage of 10 kV, a spot size of 4 nm, and a magnification of 5,000x.

##### 2.3.2.2 UV-Vis diffusive reflectance spectroscopy

The diffuse reflectance (R_d_) spectra were collected in a UV-Vis spectrophotometer (Model Evolution 220, ThermoFisher, United States). Spectralon diffuse reflectance material based on polytetrafluoroethylene (PTFE) was applied as a white reflection pattern (reflection = 100%). These measurements were made in the range of 200–1000 nm with a spectral resolution of 1 nm. The yellowness index (YI) was calculated from the reflectance measurements by Eq. 1.

$$YI=\frac{\left(R+G\right)}{B^{2}}$$ (1)

where R, G, and B are reflectance intensity at 680, 530, and 470 nm, respectively.

The optical bandgaps ( $E_{g}$ ) of the PVC samples were estimated from R_d_ data (in %) using Tauc’s plots [hν F (R_d_)]^1/n^ *versus* hν and extrapolating the linear region in the radiation energy axis (hν). h is Planck’s constant, ν is the frequency of electromagnetic radiation, n depends on the nature of the electronic transition (n is equal to two for indirect transition and to ½ for direct transition), and F (R_d_) is the Kubelka-Munk function is determined given by Eq. 2 (Li et al., 2012; Shebi and Lisa, 2019).

$$F\left(R_{d}\right)=\frac{{\left(100-R_{d}\right)}^{2}}{2R_{d}}$$ (2)

##### 2.3.2.3 Fourier-transform infrared absorption spectroscopy (FTIR)

Fourier-transform infrared spectroscopy (FTIR) with attenuated total reflectance (ATR) diamond accessory was performed on Spectrum Two equipment (PerkinElmer Inc., Massachusetts, United States). The spectra were collected with 4 cm^−1^ spectral resolution, 64 scans, from 4,000 to 500 cm^−1^. The PVC degradation was evaluated by the carbonyl (I_C=O_), polyene (I_C=C_), and hydroxyl (I_OH_) indexes using Eq. 3, according to the literature (Yousif et al., 2016).

$$I=\frac{A_{group}}{A_{1328}}$$ (3)

where $A_{1328}$ is the infrared absorbance reference peak at 1328 cm^-1^ associated with the scissoring and bending of CH_2_ groups. $A_{group}$ is the infrared absorption at 1722 (carbonyl group), 1602 (polyene), and 3,500 cm^-1^ (hydroxyl group) connected with chemical groups generated by the PVC degradation reactions.

##### 2.3.2.4 Thermogravimetric analysis (TGA)

The thermal stability of the polymeric samples was evaluated by a TGA thermal analyzer (Mettler Toledo, United States) using alumina pans. The samples were heated from 50°C to 600°C (heating rate = 10°C min^-1^) under N_2_ atmosphere (50 mL min^-1^).

##### 2.3.2.5 X-ray photoelectron spectroscopy (XPS)

The XPS high-resolution spectra were collected using K-alpha + equipment (ThermoFisher Scientific Inc., Massachusetts, United States) with monochromatic radiation A1Kα at room temperature (pass energy = 50 keV; energy step = 0.1 eV). The samples were plasma etched to perform XPS depth-profile of silver and carbon elements (ion energy = 2000 eV; raster size = 2.00 mm; depth-profile etch time = 5 s). The etched depths of the PVC samples were estimated by the etching rate of Ta_2_O_5_ standard (0.29 nm s^-1^). The XPS spectra peak-fittings were performed in CasaXPS version 2.3.25, using U 2 Tougaard background approximation and finite Lorentzian asymmetric (LF) lineshape (with relative sensitivity factors = 1). XPS spectra were calibrated to give C-C/C-H binding energy (C1s region) of 284.8 eV (Baibarac et al., 2021).

##### 2.3.2.6 Mechanical properties

Uniaxial tensile tests were performed in a Universal Testing Machine from Instron, using a load cell of 50 kN and a test speed of 1.5 mm min^−1^, according to ASTM D1708 (micro tensile). Notched Izod impact strength was measured at room temperature (25°C) by an Izod Impact Tester (Shanta Engineering, India) with a hammer pendulum of 2.71 J, following method A in ASTM 256D. All mechanical data were determined using 2-6 specimens.

##### 2.3.2.7 Antiviral assays

Surface antiviral tests were carried out in triplicate according to the ISO 21702:2019 standard. Films were cut into 5 cm^2^ squares, in laminar flow with sterile scissors, decontaminated with 70% ethanol, packed in surgical grade paper, sterilized for 20 min at 121°C in saturated steam under a pressure of 110 kPa (autoclave), and then dried in an oven at 51°C for 4 h.

Briefly, the Vero E6 cell line (ATCC–CRL1586) was cultured using Eagle’s Minimal Essential Medium (EMEM) (Sigma-Aldrich) containing 10% fetal bovine serum and 1% penicillin/streptomycin (Gibco®) incubated with 5% CO_2_ at 37°C. After culturing, the cells were transferred to a 96-well plate containing 1 × 10⁵ cells/well and incubated until reaching 80%–90% confluence. The virus inoculum used was SARS-CoV-2 (B.1.1.28 strain) 2.5 × 10⁶ TCID_50_/mL titrated according to TCID_50_ (50% Tissue Culture Infectious Doses) method. For sample contamination, the tests were carried out in a BSL-3, in a biological safety cabinet Class II B2. 100 μL of the virus inoculum were added to the center of the samples, spread with a sterile disposable loop, and incubated at room temperature (direct contact times = 30, 60, and 120 min). The material was recovered with a sterile swab and added to a Falcon tube with 0.9 mL of EMEM medium, being vortexed for 1 min 150 μL of eluate aliquots were plated on previously 80%–90% confluent VeroE6 1 × 10^4^ cells/well in triplicate, in a 96-well plate, incubated at 37°C in an oven with 5% CO_2_. After 48 h of incubation, the antiviral activity was evaluated through the cytopathic effect and cell viability by the MTT (3-[4,5-dimethylthiazol-2-yl]-2,5 diphenyl tetrazolium bromide) colorimetric assay to assess cellular viability. The results are expressed in percentage of viral inactivation (Table 1) through cell viability compared to cell controls in the presence or absence of the virus.

**TABLE 1:** Nomenclature for the antiviral activity^a^ assays.

Log reduction	Reduction factor	Inactivation percentage (%)	Activity
1	10	90	Not virucidal
2	100	99	Not virucidal
3	1,000	99.9	Not virucidal
4	10,000	99.99	Virucidal
5	100,000	99.999	Virucidal
6	1,000,000	99.9999	Virucidal

^a^ Antiviral activity: difference in the logarithm of virus infectivity titer found in an antiviral-treated product and an untreated product after inoculation and contact with the virus.

##### 2.3.2.8 Statistical analysis

One-way analysis of variance (ANOVA one-way) and Tukey’s and Dunn’s tests were applied to statistically evaluate the significant differences between the properties of the samples measured, using the GraphPad Prism 7.04 and a 95% confidence level.

## 3 Results and discussion

### 3.1 AgNP suspension

#### 3.1.1 Chemical composition

The AgNP liquid suspension has C (Kα = 0.277 keV) and O (Kα = 0.525 keV) predominantly in its composition, and a trace concentration level of Ag (Lα = 2.984 keV) was identified in the EDS spectrum (Supplementary Figure S1). Sodium (Kα = 1.041 keV) also appears in the AgNP suspension. Amadio and collaborators (Amadio et al., 2018) also identified sodium in this commercial AgNP suspension. According to the results of ICP-OES, the silver and zinc contents in the AgNP suspension are 130 ± 13 mg and 0.02 ± 0.01 mg per milliliter of AgNP suspension, respectively. Copper, another chemical element in the composition of antimicrobial agents commonly used as additives in polymers, was not identified in the antimicrobial suspension by ICP-OES.

**FIGURE 1:**
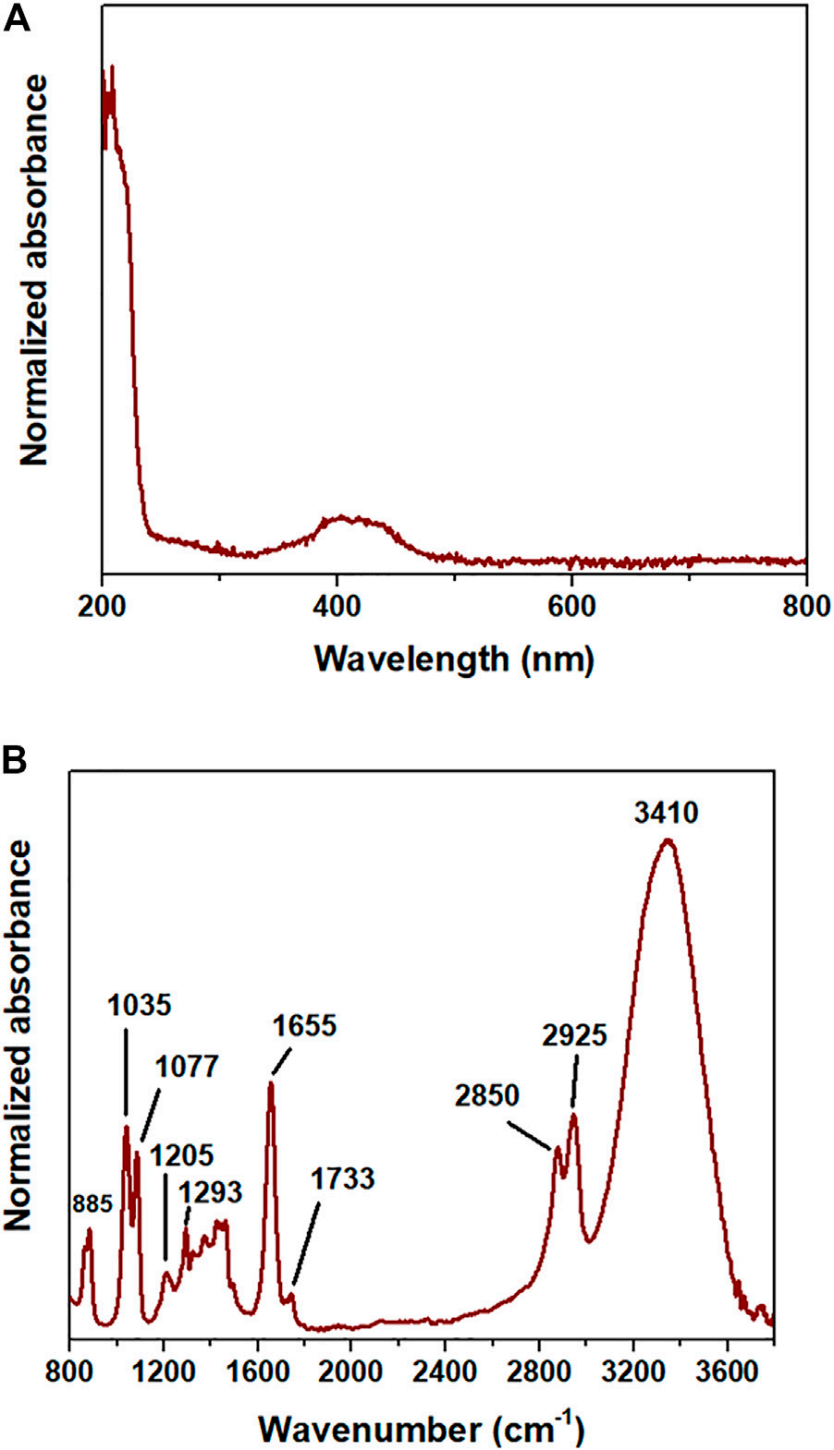
**(A)** UV-Vis spectrum and **(B)** FTIR spectrum of the AgNP suspension.

The UV-Vis absorption band (Figure 1A) in the 350–500 nm range (absorption maximum at 430 nm) is due to the AgNP surface plasmon resonance (Rehan et al., 2015; Eya’ane Meva et al., 2016). According to the literature (Poisson, 2021), this AgNP suspension is composed not just of silver (Ag) but also of ethylene glycol (C_2_H_6_O_2_), poly (vinyl pyrrolidone) (PVP), and water. Ethylene glycol and PVP act as stabilizing agents for AgNPs through a surface-coating stabilization process (Safo et al., 2019).

The FTIR spectrum of the AgNP suspension in Figure 1B presents infrared absorption bands at 860, 885, 1035, 1077, 1215, 1370, 1655, 1733, 2850, 2925, and 3,350 cm^-1^. The absorption signal at 885 cm^-1^ is related to CH_2_ wagging vibrations, and at 1215 cm^-1^ is due to the elongation of C-C bonds (aliphatic carbon) from aliphatic moieties in ethylene glycol (Saikia et al., 2017; Guo et al., 2018). The signal at 3,330 cm^-1^ may be associated with -OH groups from ethylene glycol and water. The absorption bands at 1279 cm^-1^ are related to the vibration of C-N groups on the PVP polymer chains (Safo et al., 2019). The FTIR signal at 1733 cm^-1^ indicates the presence of C=O groups of the ketone group in the pyrrolidone ring of the PVP polymer chains. The signal at 1655 cm^-1^ can be attributed to the vibrations of -OH groups and, also, to the C=O stretching from PVP. This FTIR signal is shifted due to the presence of ethylene glycol and silver in the suspension (Safo et al., 2019). The infrared signals in the FTIR spectrum located at 2925 and 2850 cm^-1^ are associated with vibrations of CH groups by asymmetric and symmetric stretching (PVP and ethylene glycol), respectively. Infrared absorption signals at 552 cm^-1^ due to stretching of Ag-O groups of AgNPs stabilized with PVP or ethylene glycol were not detected because it is outside the range of the spectrum analyzed by the ATR-FTIR equipment (Assis et al., 2021).

#### 3.1.2 AgNP particle size

**FIGURE 2:**
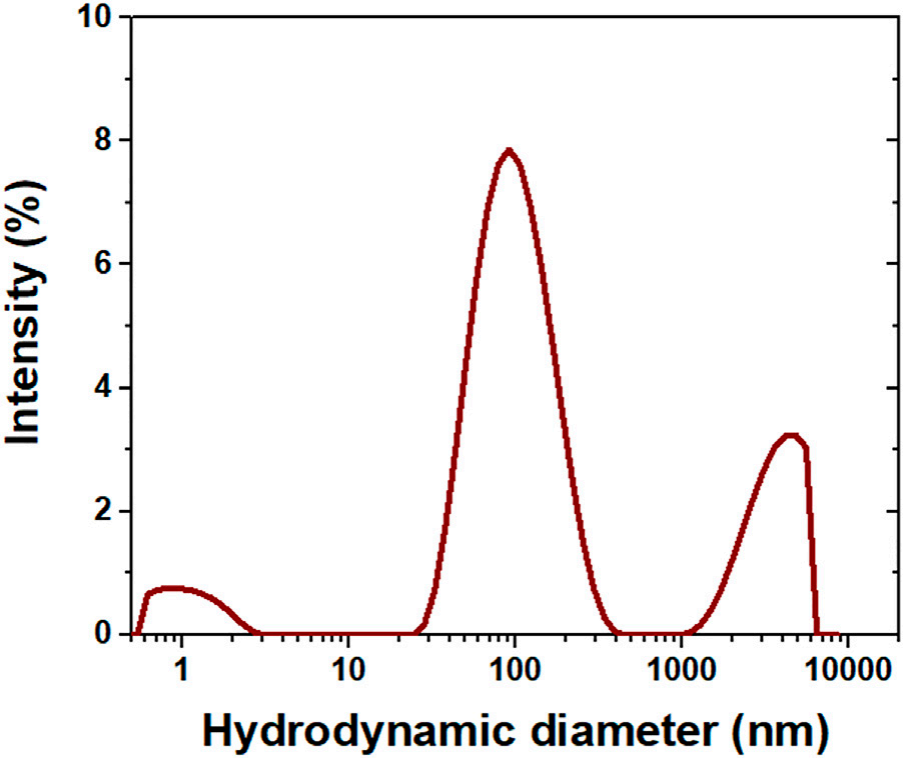
Hydrodynamic diameter distribution of the AgNP suspension.

As identified in Figure 2, the suspension presents particles with a trimodal hydrodynamic diameter distribution: less than 10 nm; between 50 and 500 nm; and greater than 1.1 µm ζ value for the AgNP suspension equals −4.7 ± 13.2 mV, indicating that the microparticles detected by DLS are associated with the aggregation of AgNPs in the suspension, which is visually yellowish and transparent. The agglomeration occurs because AgNPs have low electrostatic charges at their surfaces that are insufficient to effectively promote the repulsion between nanoparticles (Shebi and Lisa, 2019). The yellow coloration is similar to the coloration of AgNP suspensions synthesized by different methods reported in different studies in the literature (Rodríguez-León et al., 2013; Kavuličová et al., 2018).

### 3.2 PVC/AgNP nanocomposites

#### 3.2.1 Scanning electron microscopy (SEM)

**FIGURE 3:**
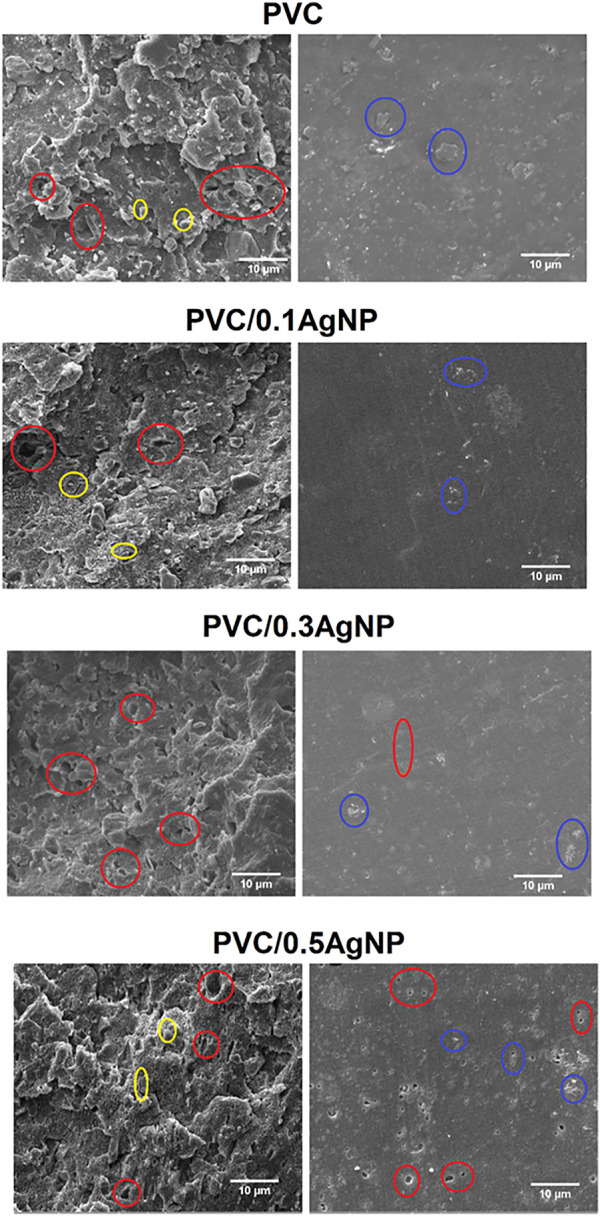
SEM images of the PVC and PVC/XAgNP nanocomposites, where X corresponds to the AgNP content (wt%). Images were obtained from the cryofractured internal surfaces (left) and external surfaces (right) of the test specimens. Different defects are highlighted in circles: cavities (red), particles at the exterior surface (blue), and interfacial voids (yellow).

Figure 3 presents SEM images for PVC and the PVC/AgNP nanocomposites. According to the supplier, the PVC presents well-dispersed microparticles of calcium carbonate (CaCO_3_) and titanium dioxide (TiO_2_). CaCO_3_ is an inorganic material widely applied in the polymer industry as a filler to reduce the cost of products based on commodity thermoplastics (Rocha et al., 2018; da Silva et al., 2021a). TiO_2_ is extensively utilized in the polymer industry as a white pigment and UV-blocking additive to hamper polymer degradation occasioned by UV exposition (da Silva et al., 2018). Still, TiO_2_ also displays photocatalytic properties suitable for self-cleaning coatings on several materials. The usage of solid particles also contributes to diminishing the plasticizer diffusion and migration to the PVC surface and external environment, which leads to substantial changes in the mechanical performance of PVC, in the case of plasticized products (Xiong et al., 2016). Microcavities and interfacial voids are identified at the cryofractured internal surface of the PVC sample due to low adhesion between the filler and polymeric matrix. Moreover, CaCO_3_ microparticles are visible at the external surface of PVC, which can cause excessive surface roughness of the PVC parts (Figure 3).

The addition of AgNP suspension to the PVC leads to the formation of several microvoids at the external surface, as can be seen on the SEM images of the PVC/0.3AgNP and PVC/0.5AgNP nanocomposites (Figure 3). It occurs due to the evaporation of volatile compounds in the AgNP suspension, which is caused by heating during the thermal processing of the PVC samples. AgNP suspension also seems to increase the number of microvoids at the cryofractured internal surface of the PVC/1AgNP nanocomposite, suggesting a more significant detachment of the CaCO_3_ particles from the PVC matrix that is justified by the poor interfacial adhesion between the phases (da Silva et al., 2018). Even knowing the size of the AgNP and AgNP aggregates in the antimicrobial silver suspension by DLS, it is impossible to identify them in the SEM images due to the low concentration of AgNP.

The SEM images in Figure 3 also show that the fracture of the PVC matrix changes from brittle to ductile due to the increase in AgNP content. This result may be associated with the small organic molecules in this silver suspension that increase the polymer chain mobility in the composite even with the presence of a micrometric filler, acting as a plasticizing agent and enabling more plastic deformation in the PVC matrix.

#### 3.2.2 UV-vis diffusive reflectance spectroscopy

**FIGURE 4:**
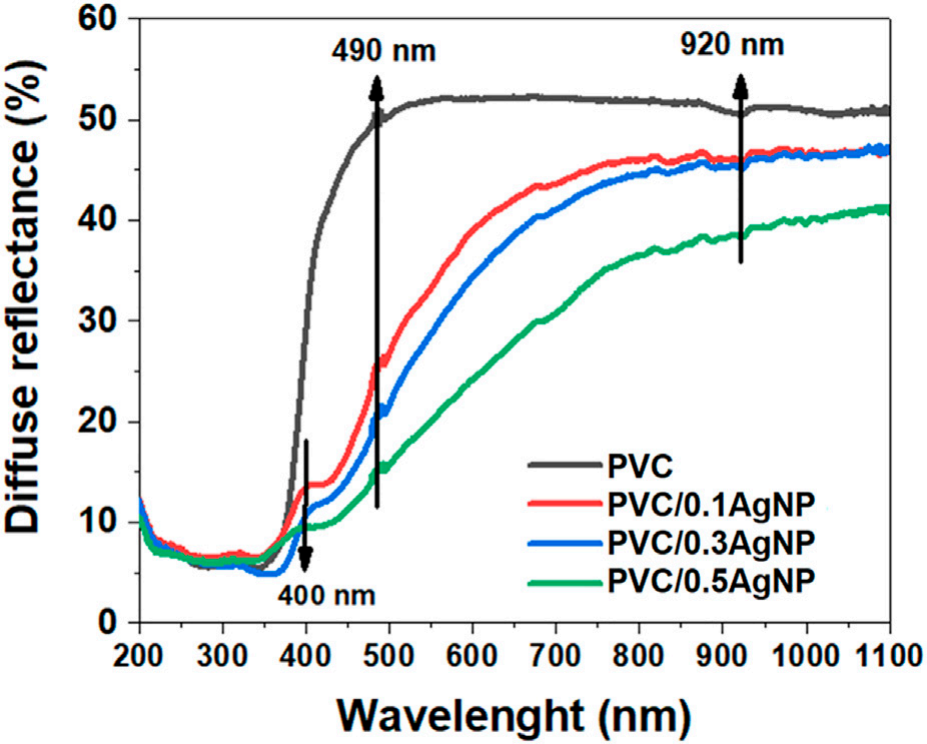
Diffuse reflectance (R_d_) spectra of the PVC and PVC/XAgNP nanocomposites (X corresponds to the AgNP content in wt%).

The diffuse reflectance (R_d_) spectra of the PVC and the nanocomposite samples are shown in Figure 4. PVC and all nanocomposites present an anomalous light dispersion at 490 nm due to an abrupt and concomitant increase in the absorptivity and refractive index of the PVC system. This phenomenon is called the Eststrahlen effect, which is associated with a predominant Fresnel reflectance over the Kubeika-Munk reflectance at this specific wavelength (Blitz, 1998; Tao et al., 2015). The PVC system displays an absorption signal at 920 nm in the near-infrared (NIR) wavelength region, in which its signal intensity is reduced as the AgNP content rises in PVC. The reason for this spectral phenomenon at 920 nm is out of our awareness, but it can be connected to the lamp change at 350 nm of the UV-Vis equipment (from deuterium to halogen lamp) during the measurements. The R_d_ intensity also decreases with the increase of the AgNP concentration in the PVC.

It is reported that PVC exhibits absorption maxima at 280 and 245 nm in the UV region due to π–π* electronic transitions in the polymer backbones (Abdel-Fattah et al., 2019). However, the PVC used in this work presents a sharp absorption profile after 450 nm, and the R_d_ intensities are minimal at 350 nm. This UV-visible absorption profile is similar to CaCO_3_/TiO_2_ hybrid particles, which are utilized commercially as an alternative white pigment to work around the higher price of TiO_2_ pigment (more expensive than CaCO_3_) and the scarcity of titanium resources (Sun et al., 2018).

According to Figure 4, the addition of AgNPs in the PVC leads to a broadening of the R_d_ intensity for higher wavelengths (redshift) occasioned by an enhancement of the absorption coefficient in the visible wavelength. The low bandgaps of the inorganic components in the PVC/AgNP nanocomposites are responsible for these results since they have optical bandgaps ( $E_{g}$ ) inferior to that of PVC. It is well known that silver and other noble metals reduce the optical bandgap of semiconducting metallic oxides, improving their UV-visible light absorption due to the introduction of lower energetic levels in the electron energy band structure of the semiconductor (Abbad et al., 2020). Moreover, the absorption of visible radiation by the AgNP plasmon resonance states with low-energetic levels must contribute to the absorption broadening in the UV-visible electromagnetic region, including the reduction of the direct ( $E_{g}^{d}$ ) and indirect ( $E_{g}^{i}$ ) optical bandgaps of the PVC and PVC/AgNP nanocomposites obtained from Tauc’s plot and Kubelka-Munk transformation here (Supplementary Figure 2S).

$E_{g}^{d}$ and $E_{g}^{i}$ values are detailed in Table 2. The optical bandgaps are close to those experimentally observed for TiO_2_ that displays bandgap around 3.2 and 2.9 eV for anatase and rutile phases, respectively (Ivanova et al., 2016; Munir et al., 2019). The anatase phase has an indirect bandgap, while the rutile presents direct electronic transitions (Ramos Jr et al., 2017). Abdel-Fattah et al. (2019) reported the direct and indirect optical bandgaps of PVC film around 4.2–4.3 eV, which are values higher than those experimentally observed in our PVC system. According to Ghadam et al. (Ghadam and Idrees, 2013), calcite (CaCO_3_) is an indirect bandgap semiconductor with $E_{g}^{i}$ very close to 5.8 eV. Then, all this information from the literature indicates that the bandgap data of the PVC in Table 2 are relative to TiO_2_. Also, increasing AgNP in the PVC nanocomposites slightly reduces the $E_{g}^{d}$ and $E_{g}^{i}$ of this oxide, as expected and explained previously (Abbad et al., 2020; Gogoi et al., 2020). Antagonistically, the yellowness index (YI) of the PVC enhances as the AgNP content increases in the PVC/AgNP nanocomposites due to the characteristic yellow color of the AgNP suspension. The YI values calculated here are coherent with the yellowish coloring aspect of the PVC samples visually observable in Table 2.

**TABLE 2:** The visual aspect, yellowness index (YI), direct ( $E_{g}^{d}$ ) and indirect ( $E_{g}^{i}$ ) optical bandgaps of the PVC and PVC/XAgNP nanocomposites (X corresponds to the AgNP content in wt%).

Sample	Visual aspect	YI (%)	$E_{g}^{d}$ (eV)	$E_{g}^{i}$ (eV)
PVC	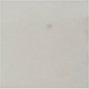	4.4	3.1	3.0
PVC/0.1AgNP	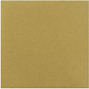	16.8	2.6	2.2
PVC/0.3AgNP	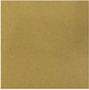	23.0	2.5	1.8
PVC/0.5AgNP	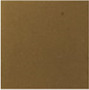	28.6	2.5	1.5

#### 3.2.3 Fourier-transform infrared absorption spectroscopy (FTIR)

**FIGURE 5:**
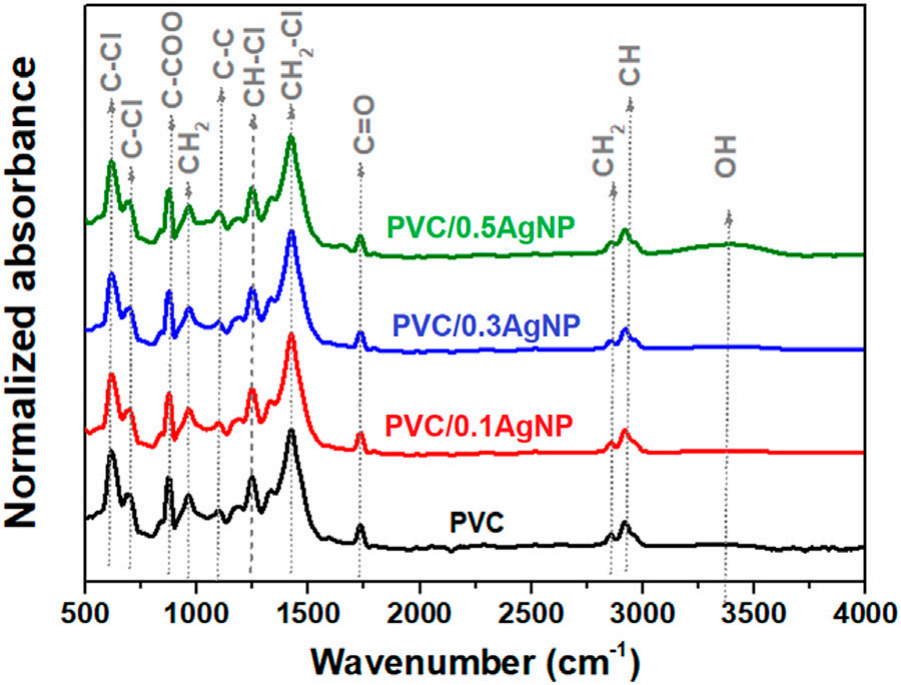
FTIR spectra from the PVC and PVC/XAgNP nanocomposites (X corresponds to the AgNP content in wt%).

Figure 5 presents the FTIR spectra of the PVC samples. There are infrared absorption signals associated with molecular vibrations of distinct chemical functional groups from PVC (Coltro et al., 2013; Park et al., 2018): C-Cl (stretching, 610 and 695 cm^-1^), CH_2_ (asymmetrical stretching, 2851 cm^-1^), C-C (stretching, 1100 cm^-1^), CH-Cl (out-of-plane angular deformation, 1253 cm^-1^), CH_2_–Cl (angular deformation, 1425 cm^-1^).

**FIGURE 6:**
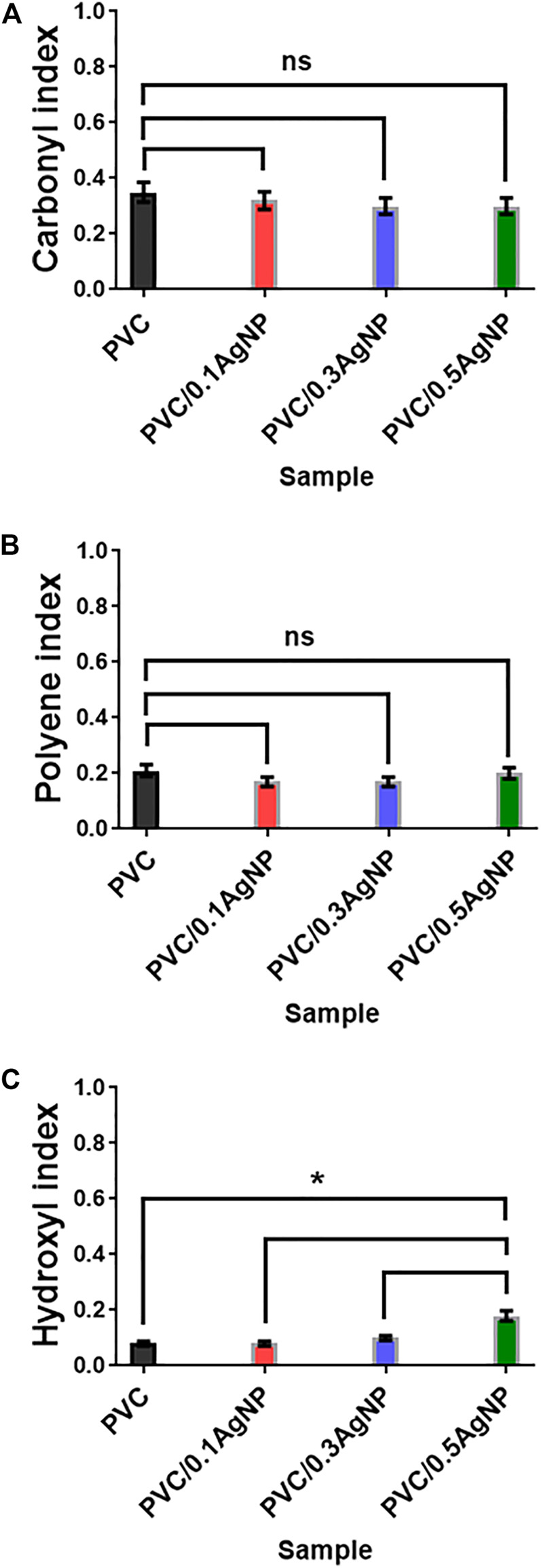
**(A)** Carbonyl (I_C=O_), **(B)** polyene (I_C=C_), and **(C)** hydroxyl (I_OH_) indexes from FTIR spectra of the PVC and PVC/XAgNP nanocomposites (X corresponds to the AgNP content). Statistical analyses are for each sample group, using Tukey’s multiple comparison tests. ns = data are not significantly different (*p*-value >0.05).

The FTIR signal at 870 cm^-1^ is associated with the C-COO bond, confirming the presence of calcium carbonate (CaCO_3_) as indicated by SEM images. Moreover, there are infrared absorption signals from carbonyl (1722 cm^-1^) and polyene (1602 cm^-1^) groups due to PVC thermooxidative degradation (Yousif et al., 2016). PVC degrades mainly by dehydrochlorination, releasing HCl with the generation of polyenes. However, chloroketones and aliphatic ketones also can be formed by alternative degradation reaction mechanisms of this polymer with oxygen gas in the atmosphere (Yousif et al., 2016; Yu et al., 2016). Also, the polyenes can suffer crosslinking reactions *via* Diels–Alder condensation, generating C=C bonds in cyclic compounds (Morikawa, 2014). As shown in Figures 6A, B, the carbonyl (I_C=O_) and polyene (I_C=C_) indexes have no significant differences, indicating that the addition of the AgNP suspension did not intensify the PVC degradation during thermomechanical mixing.

PVC photooxidation reactions due to UV irradiation lead to polymer chain scissions with increasing hydroxyl groups in the polymer (3,500 cm^-1^) (Yousif and Hasan, 2015). However, the hydroxyl index (I_OH_) from the PVC (Figure 6C) was significantly enhanced by adding 0.5 wt% of AgNP, which is expected by the presence of OH groups from components in the silver suspension (PVP and ethylene glycol).

#### 3.2.4 X-ray photoelectron spectroscopy (XPS)

**FIGURE 7:**
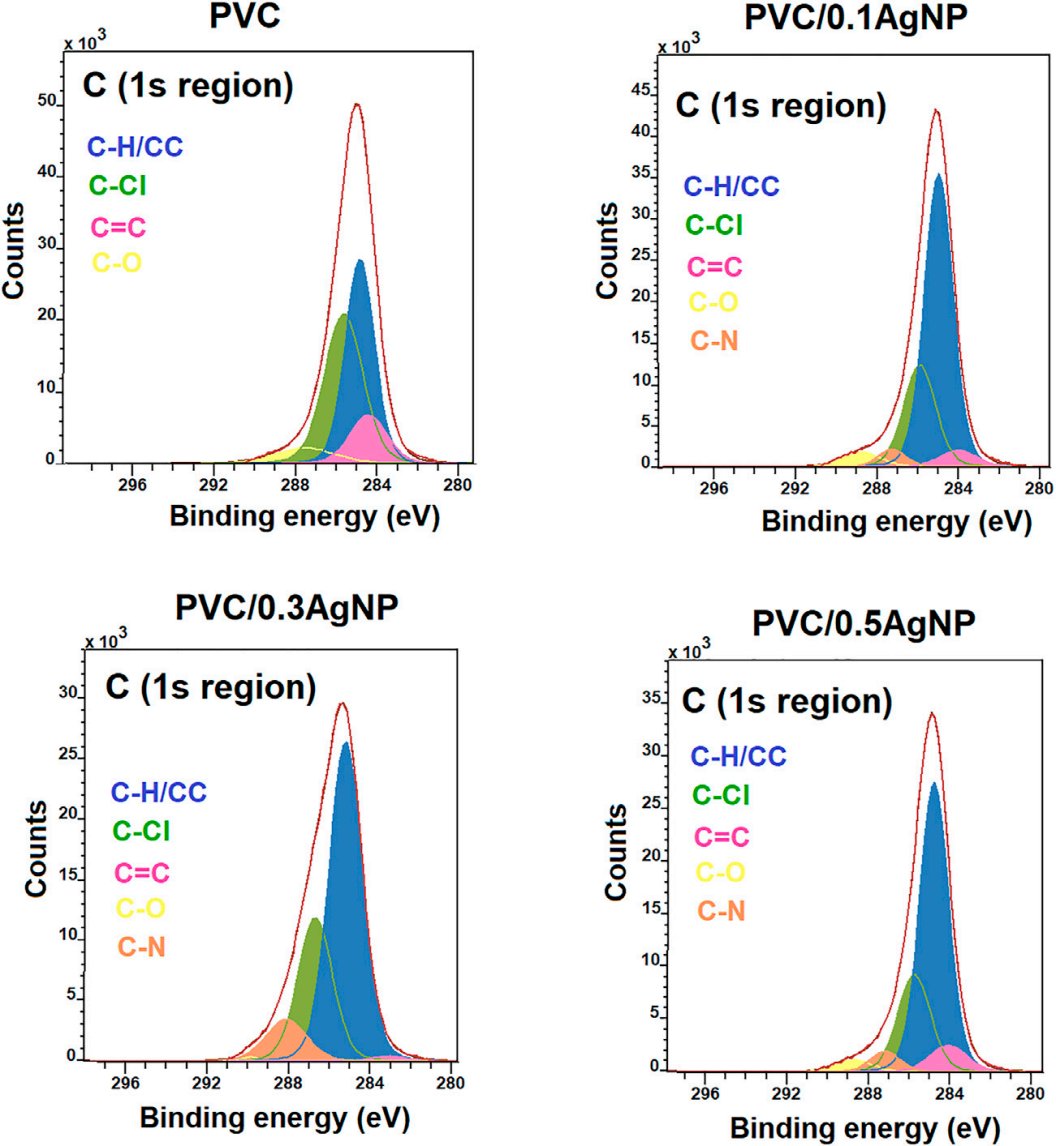
C1s XPS high-resolution spectra from the PVC and PVC/*X*AgNP nanocomposites (*X* corresponds to the AgNP content in wt%).

The XPS spectra of binding energies for carbon bonds (C1s XPS region) in PVC samples are shown in Figure 7. Four C1s fitting peaks are identified in PVC at 284.8 eV (C–C/C–H), 286.3 eV (C–Cl), 284 eV (C=C), and 288.1 eV (C-O) (Wang et al., 2015; Fu et al., 2019; Baibarac et al., 2021). The PVC nanocomposites present another XPS peak at 287.5 eV from C-N bonds, shifting the XPS signal from C-O bonds to 289–290 eV. The C-N bonds are associated with stabilizer compounds in the AgNP suspension, such as poly (vinyl pyrrolidone) (PVP). The presence of C=C bonds at the surface of the PVC samples corroborates the FTIR and UV-Vis data that indicate PVC degradation.

**FIGURE 8:**
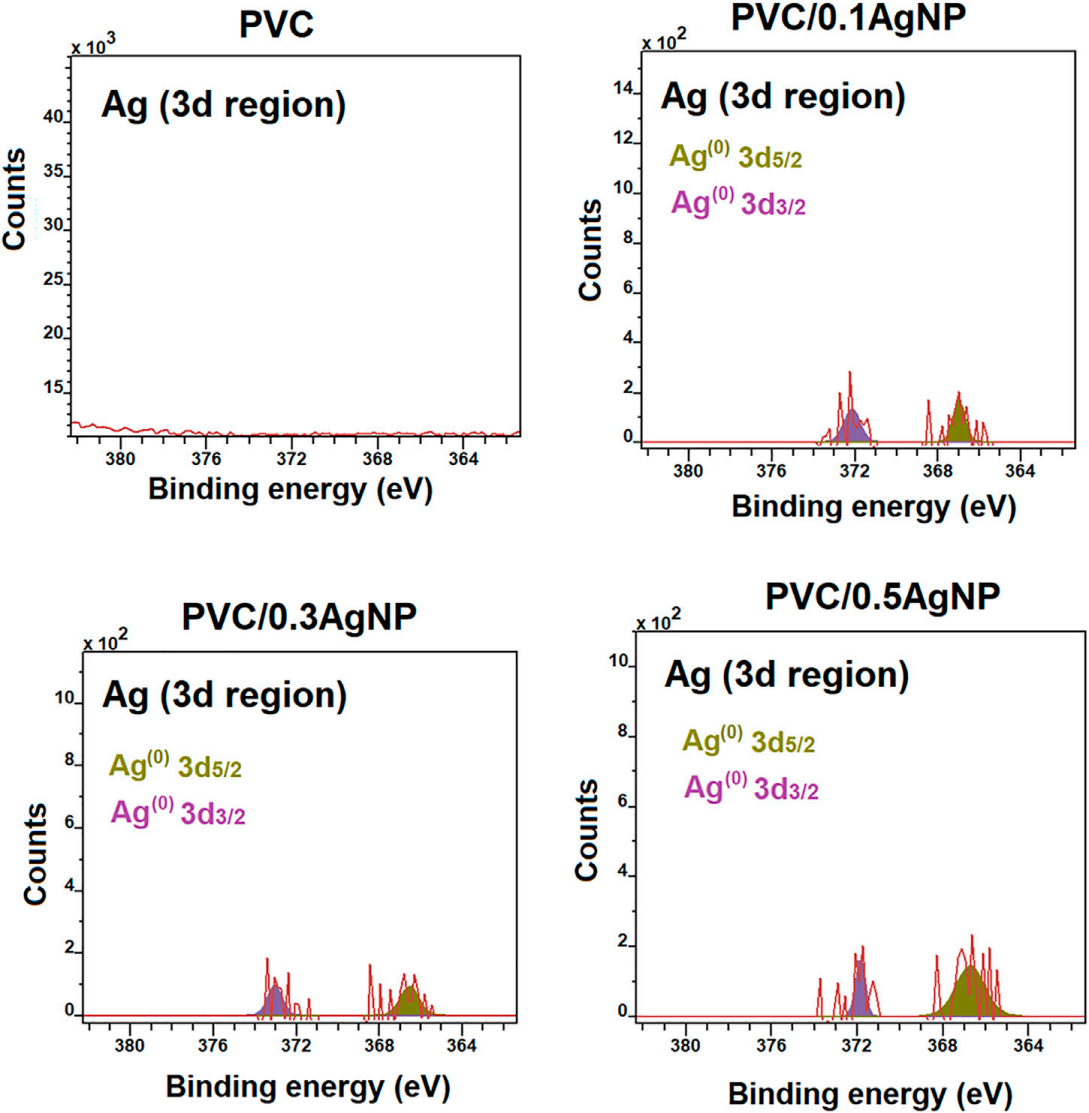
Ag3d XPS high-resolution spectra from the PVC and PVC/*X*AgNP nanocomposites (*X* corresponds to the AgNP concentration).

The Ag3d XPS spectra (Figure 8) confirm the presence of Ag (0) (metallic silver) in the PVC nanocomposites due to the presence of XPS signal peaks at 372.2 (Ag3d_3/2_) and 365.5 eV (Ag3d_5/2_) (Sharma et al., 2018). The low intensity of Ag (0) signal can be associated with the attenuation of electrons caused by the capping effects of the AgNPs by PVP and ethylene glycol (Binaymotlagh et al., 2022).

The XPS depth-profile results in Supplementary Figure S3 indicate that the PVC sample undergoes a more pronounced dehydrochlorination degradative process at the surface than the PVC nanocomposites, since the C-Cl peak area increases while the C=C peak area reduces along the sample depth. The silver suspension seems to ease the localized thermooxidative degradation at the PVC surface due to the local heating during the molding. Moreover, the XPS depth-profile data suggest that silver nanoparticles are distributed within the PVC/AgNP nanocomposites, which is essential to their antimicrobial performance in applications where the surface is subject to constant wear to maintain the AgNP content at the PVC nanocomposites consistently higher than the minimum antimicrobial concentration.

#### 3.2.5 Mechanical properties

**FIGURE 9:**
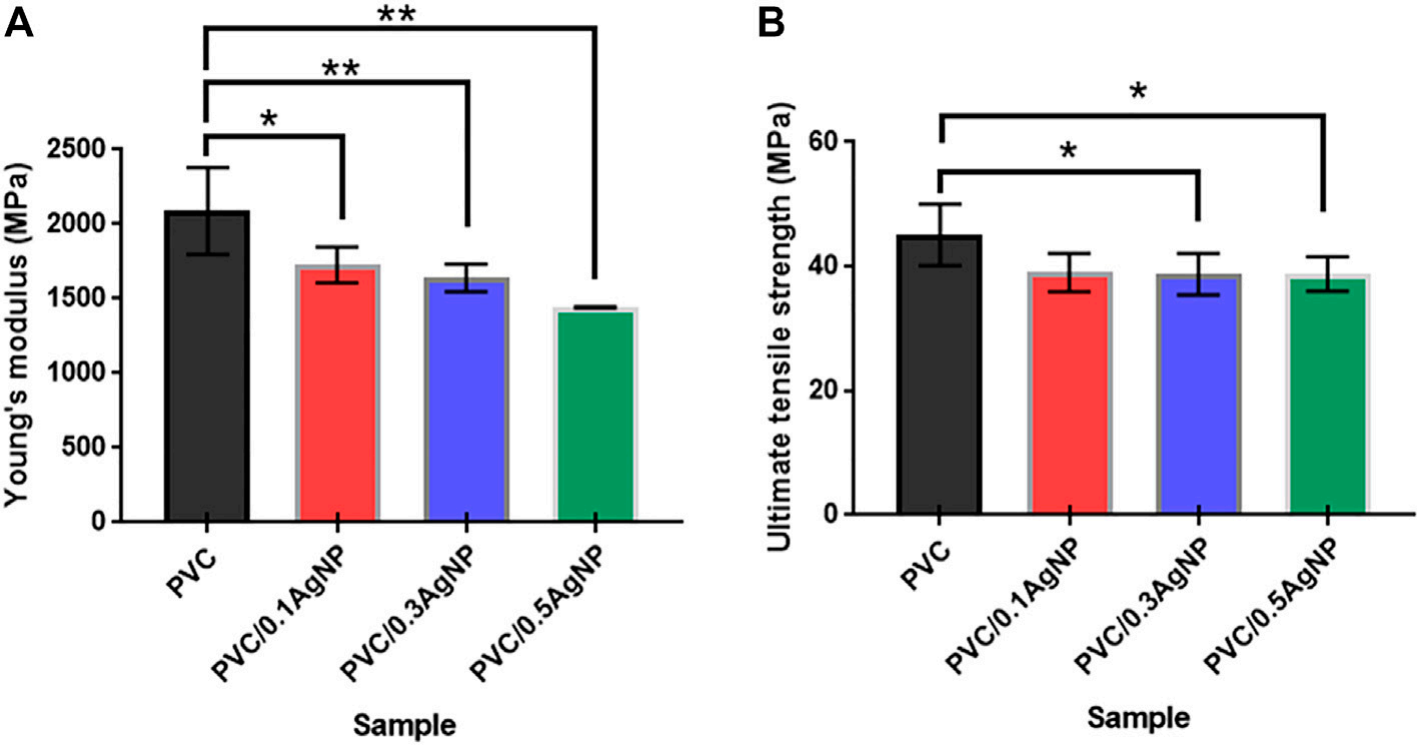
**(A)** Young’s modulus and **(B)** ultimate tensile strength of the PVC and PVC/XAgNP nanocomposites, where X corresponds to the AgNP content. The data represent mean ± standard deviation (SD) (n = 3–6). Statistical analyses are for each sample group, using Tukey’s multiple comparison tests. The *p*-value is considered significant at <0.05 (95% confidence level). ***p < 0.001, **p < 0.01, and *p < 0.05 indicate mean data significantly different.

Young’s modulus (E) and ultimate tensile strength (σ_max_) from uniaxial tensile tests of the PVC and PVC/AgNP nanocomposites are shown in Figure 9. PVC had a tensile strength of 45.1 ± 4.9 MPa and a tensile modulus of 2.1 ± 0.3 GPa. The PVC and all composites were tested at the same ASTM standard and strain rates, enabling a direct comparison of the uniaxial tensile measurements. For this purpose, we applied Tukey’s multiple comparison test as ANOVA one-way method where the results are considered significantly different if the *p*-value is lower than 0.05 using a 95% confidence level. The PVC nanocomposites present Young’s moduli equal to 1.7 ± 0.1, 1.6 ± 0.1, and 1.4 ± 0.1 GPa when added 0.1, 0.3, and 0.5 wt% of AgNP in the PVC, respectively. According to ANOVA, Young’s moduli of the PVC/AgNP nanocomposites are identical. However, they are significantly lower than the E value of the PVC, probably due to the local plasticizing effect of the AgNP suspension on the PVC observed in the SEM images *via* lubricant or gel swelling mechanisms (Daniels, 2009; Quesada-Pérez et al., 2011; Langlois and Deville, 2014). Consequently, the immobilization of the polymer matrix due to inorganic particle stiffness does not contribute significantly to the enhancement of Young’s modulus of the PVC (Watt et al., 2020).

The ultimate tensile strengths of the PVC/AgNP nanocomposites are also statistically equal, independently of the AgNP content, as shown in Figure 9B. However, the σ_max_ data from the PVC is slightly superior to those from PVC/0.3AgNP and PVC/0.5AgNP nanocomposites (around 32–41 MPa), confirming the hypothesis that the AgNP suspension reduces the adhesion between the PVC matrix with the inorganic microparticles as suggested by SEM analysis. This reduction in the interfacial adhesion leads to a poor stress transfer between these phases, causing a decrease of σ_max_ for the composite.

**FIGURE 10:**
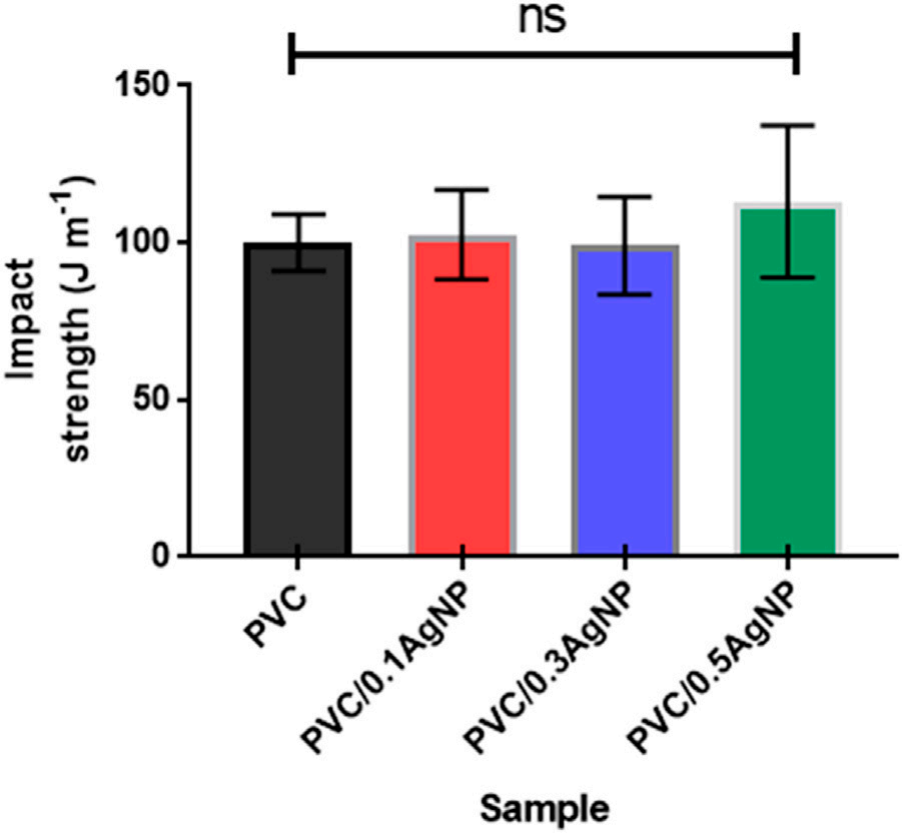
Izod impact strength of the PVC and PVC/XAgNP nanocomposites, where X corresponds to the AgNP content (wt%). The data represent mean ± SD (n = 5). Statistical analyses are for each sample group, using Tukey’s multiple comparison tests. ns = data are not significantly different (*p*-value >0.05).

The toughness of the PVC samples was evaluated by Izod impact tests, and the results are shown in Figure 10. There is no significant difference in impact strength for PVC with the increase of the AgNP concentration, despite the increase of cavities on the PVC surface caused by the insertion of AgNPs. This result is important, as the antimicrobial grade PVC must have similar toughness to the original commercial PVC compounds used to produce parts for hospital environments. The impact strength results are 100 ± 9 (PVC), 103 ± 14 (PVC/0.1AgNP), 99 ± 16 (PVC/0.3AgNP), and 113 ± 24 J m^-1^ (PVC/0.5AgNP). In another way, the decrease in strength and toughness of PVC/AgNP nanocomposites due to the presence of microstructural defects caused by AgNPs was reported by Merchan et al. (2010). Braga et al. (2019) observed a similar reduction of mechanical strength of the PVC films (prepared by solvent casting method) caused by AgNP aggregation. According to them, the silver nanoparticles at concentrations of 2 – 8 wt% generated a less cohesive internal structure, affecting mechanical strength and also decreasing the elongation at the break of the PVC (Braga et al., 2019).

#### 3.2.6 Thermogravimetric analysis (TGA)

**FIGURE 11:**
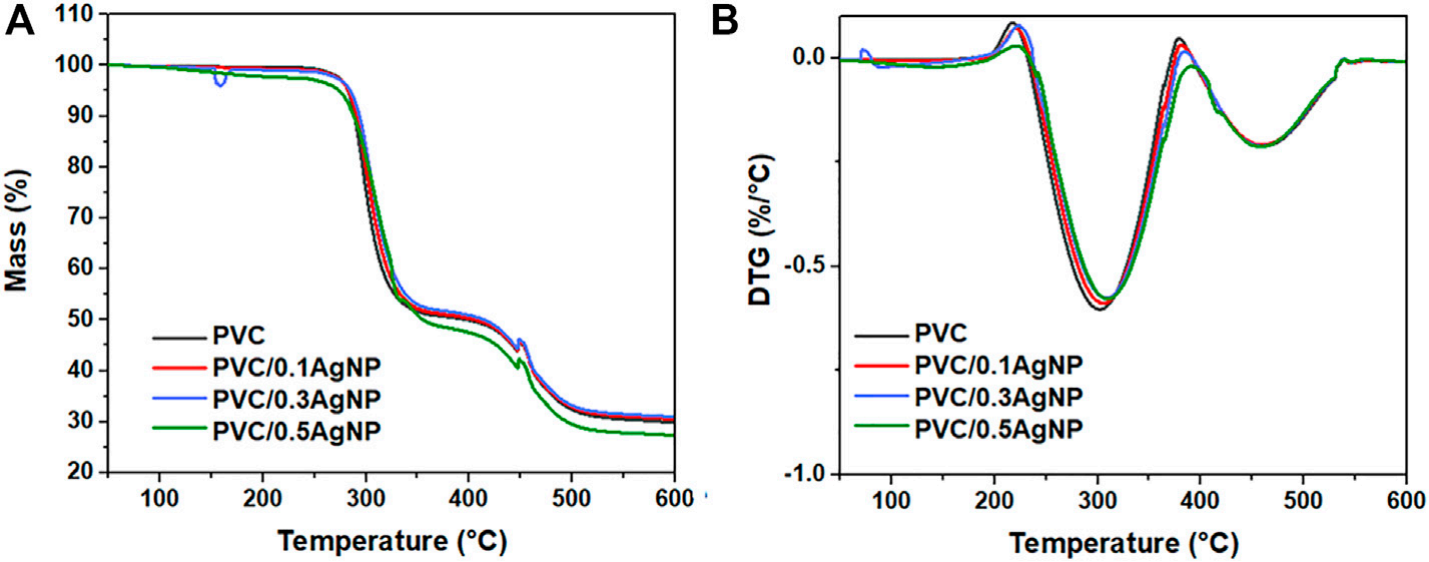
TGA thermograms **(A)** and the DTG curves **(B)** from the PVC and PVC/XAgNP nanocomposites, where X corresponds to the AgNP content (wt%).

Figure 11 presents the thermal decomposition profiles of the polymeric samples. PVC thermally decomposes *via* two distinct stages. From 250°C to 350 °C, the major mass loss (50 wt%) occurs due to the PVC dehydrochlorination with the formation of polyene sequences along the PVC polymer backbone (Kayyarapu et al., 2016). The second stage, from 420°C to 550 °C, involves the mass loss of around 20 wt% associated with the decomposition of the polyene sequences, generating carbonaceous residues (Cruz et al., 2021) that remain along with the inorganic particles (identified by SEM).

All PVC samples have similar onset thermal decomposition temperatures ( $T_{onset}$ ), varying from 276°C to 289 °C, as detailed in Table 3. From the DTG curves, the temperatures at the maximum thermal decomposition rate ( $T_{\max}$ ) of each stage were determined. There is a slight rising on the $T_{\max}$ average values from the PVC’s first thermal decomposition step due to the increase in the AgNP content. The silver nanoparticles may hamper the loss of volatiles generated by the PVC dehydrochlorination reactions during its heating. $T_{\max}$ is associated with the PVC second thermal decomposition step and is not affected by the AgNP content. Shimoga et al. (2019) observed the opposite effect of AgNP concentration on $T_{onset}$ for the first thermal decomposition step in AgNP/PVC films obtained by casting. They attributed the low thermal stability of AgNP/PVC films to the solvent molecules trapped between the polymer chains that caused thermal decomposition of PVC at temperatures below 200°C (Shimoga et al., 2019). Furthermore, adding 0.5 wt% of AgNPs provided the highest total weight loss for the nanocomposites at 600°C, which is justified by the higher amount of low-mass organic compounds in the AgNP suspension that are thermally decomposed in the PVC matrix above 400 °C. Braga et al. (2019) also observed that the dehydrochlorination onset temperature of AgNP/PVC films decreases with the enhancement of AgNP concentration, but it causes a reduction of the total weight loss since the content of inorganic materials increases in the PVC matrix.

**TABLE 3:** $T_{onset}$ and $T_{\max}$ temperatures from TGA and DTG measurements of the PVC samples.

Sample	$T_{onset}$ (°C)	$T_{\max}$ (°C)
PVC	281 ± 5	302 ± 75
		460 ± 78
PVC/0.1AgNP	282 ± 5	307 ± 75
		460 ± 78
PVC/0.3AgNP	283 ± 5	311 ± 75
		460 ± 78
PVC/0.5AgNP	284 ± 5	311 ± 75
		460 ± 78

#### 3.2.7 Antiviral assays

The assay was performed at different contact times, with 30, 60, and 120 min (Supplementary Figure S4a). The percentage of viral inactivation observed through cell viability increases with longer contact times. PVC/0.3AgNP and PVC/0.5AgNP samples present virucidal activity compared to cell controls with significant differences against the SARS-CoV-2 positive control, according to Dunn’s tests (Table 4; Supplementary Figure S4a). Therefore, to achieve an inactivation percentage of 99.99% in 48 h, the PVC must contain at least 0.3 wt% of AgNPs. The antiviral activities from these nanocomposites were evidenced by the decrease in the cytopathic effects caused by the virus that reduced the percentage of viable cells. There are few published works on the antiviral activity of polymer matrix nanocomposites against SARS-CoV-2 variants. Lam et al. (2022) reported that polyurethane/AgNP nanocomposites could reduce the amount of SARS-CoV-2 beta (B.1.351) virions by 67% within 24 h of direct contact antiviral assays. According to TCID_50_ reduction assays, Assis et al. (2022) observed that SARS-CoV-2 antiviral activity of propylene (PP) composites with 0.3 wt% of Ag_2_XO_4_ (X = W, Mo, and Cr).

**TABLE 4:** Antiviral activity results from the PVC and PVC/XAgNP nanocomposites.

Sample	Log reduction	Inactivation percentage (%)	Activity
PVC	1	90	Not virucidal
PVC/0.1AgNP	2	99	Not virucidal
PVC/0.3AgNP	4	99.99	Virucidal
PVC/0.5AgNP	4	99.99	Virucidal

^a^ Results are expressed as a viral inactivation percentage through cell viability compared to cell controls in the presence or absence of SARS-CoV-2.

The time-dependent virucidal effect of the samples is directly associated with increased contact time due to the longer exposure time of virions to AgNPs, Ag^+^ ions, and ROS that cause irreversible damage to viral particles (da Silva et al., 2021b). Jeremiah et al. (2020) also observed time-dependent virucidal effects of AgNP suspensions against SARS-CoV-2 virions.

## 4 Conclusion

In this work, PVC/AgNP nanocomposites were successfully prepared *via* melt mixing, which is a suitable route for the large-scale production of polymeric products with large sizes and complex geometries. SEM images evidence the formation of surface defects on the PVC due to the addition of AgNPs, leading to changes in Young’s modulus and ultimate tensile mainly when the AgNP content is higher than 0.1 wt%. The toughness of the PVC/AgNP nanocomposites is similar to the PVC. TGA and FTIR data indicate that the AgNPs do not lead to significant degradation of the PVC matrix bulk. According to the XPS high-resolution depth-profile measurements, the AgNP suspension prevented located dehydrochlorination degradation of the PVC matrix at the surface of the PVC/AgNP nanocomposites.

UV-Vis spectroscopy evidences an increase in the PVC’s yellowness index (YI) due to the increased AgNP content, causing visual changes inherent to the compounds with yellow color in the AgNP suspension. The cytopathic effect and cell viability assays proved that the nanocomposites present virucidal activity against SARS-CoV-2 within 48 min if the AgNP content is at least 0.3 wt%. The antiviral nanocomposites seem adequate for application on plastic objects to reduce the transmission of COVID-19, mainly in environments with high biological risks of exposure to transmitting viral diseases through contact with contaminated surfaces, such as hospitals and medical clinics.

## Data Availability

The original contributions presented in the study are included in the article/Supplementary Material, further inquiries can be directed to the corresponding authors.
