# Supplementary material for: PVC containing silver nanoparticles with antimicrobial properties effective against SARS-CoV-2
Source: Front Chem. 2023 Mar 13;11:1083399. doi: 10.3389/fchem.2023.1083399 (PMC10042293; doi:10.3389/fchem.2023.1083399)
Supplement: Supplementary file 1 [file DataSheet1.docx]

(Supplementary Material)

**PVC containing silver nanoparticles with antimicrobial properties effective against SARS-CoV-2**

Daniel J. da Silva^1^, Guilherme B. Gramcianinov^1^, Pamela Z. Jorge^1^, Vanessa B. Malaquias^2^, Augusto A. Mori^2^, Mário H. Hirata^2^, Sergio A. M. Lopes^3^, Luciano A. Bueno^1^, Mathilde Champeau^1^, Danilo J. Carastan^1*^

^1^ Center for Engineering, Modeling, and Applied Social Sciences (CECS), Federal University of ABC (UFABC), Av. dos Estados, 5001, Santo André, CEP 09210-580, SP, Brazil.

^2^ Department of Clinical and Toxicological Analysis, Faculty of Pharmaceutical Sciences, University of São Paulo, Av. Professor Lineu Prestes, 580, São Paulo, CEP 05508-900, SP, Brazil.

^3^ BRGoods Indústria e Comércio de Produtos Hospitalares, R. Soldado Antonio Lopes Pereira, 240, Indaiatuba, CEP 13346-610, SP, Brazil.

****E-mail addresses:*** mathilde.champeau@ufabc.edu.br (M. Champeau), [danilo.carastan@ufabc.edu.br](mailto:danilo.carastan@ufabc.edu.br) (D.J. Carastan)

# ICP-OES

Table 1S. ICP-OES operating parameters for silver, copper, and zinc quantification.

| **Plasma Parameter** | **Condition** |
| --- | --- |
| Radiofrequency power | 1 kW |
| Plasma gas flow | 15 L min^-1^ |
| Auxiliary gas flow | 1.5 L min^-1^ |
| Nebulization pressure | 200 kPa |
| Replicate read time | 5 |
| Stabilization delay | 15 s |
| Sample uptake delay | 15 s |
| Pump rate | 15 rpm |
| Rinse time | 10 s |
| **Chemical element** | **Spectral line** |
| Ag | 328.068 nm |
| Cu | 327.395 nm |
| Zn | 213.857 nm |

# EDS


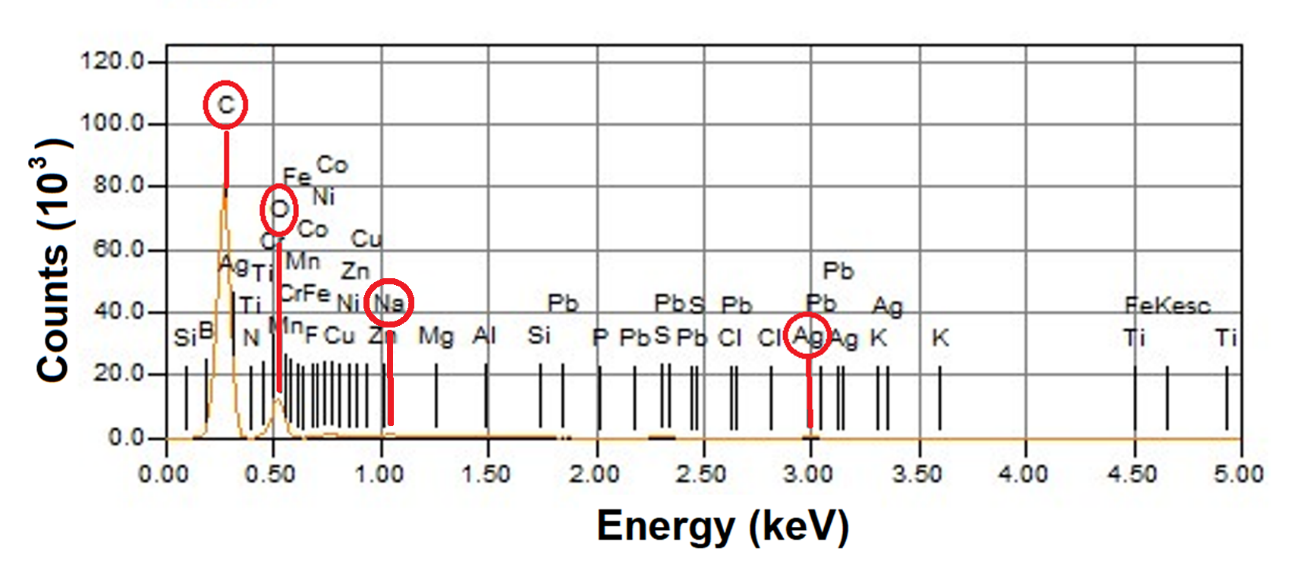
Figure 1S. EDS spectrum of the AgNP suspension. Chemical elements identified in the EDS spectrum were highlighted with red circles.

# UV-Vis spectroscopy


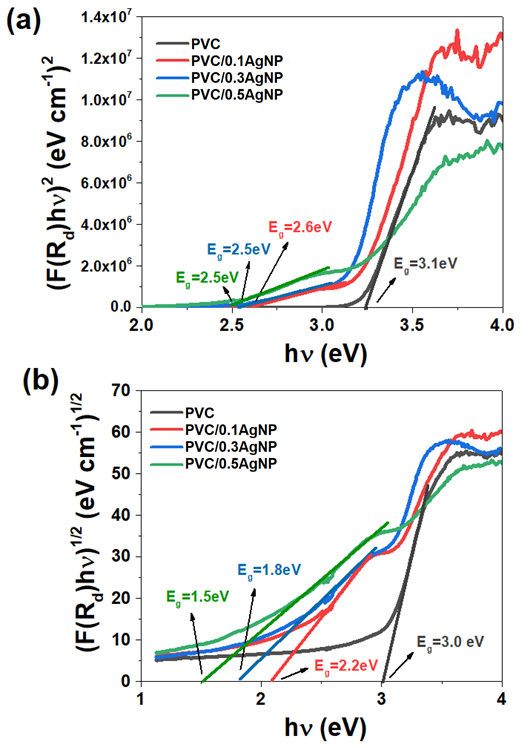


Figure 2S. Tauc’s plot for the direct (a) and indirect (b) electronic transitions in the PVC and PVC/*X*AgNP, where *X* corresponds to the AgNP content.

# XPS spectroscopy


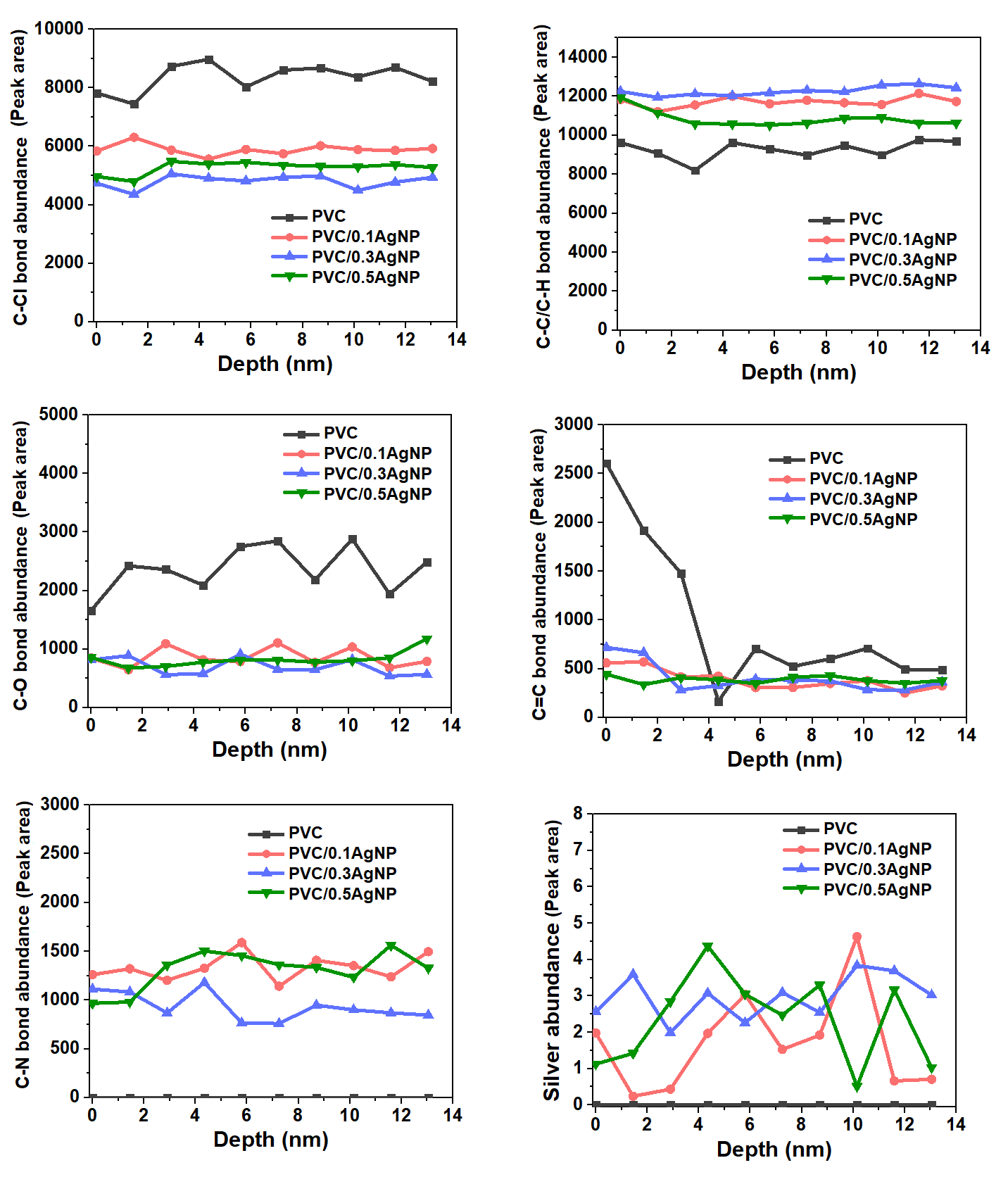


Figure 3S. XPS depth-profile for silver and carbon chemical bonds from the PVC and PVC/*X*AgNP, where *X* corresponds to the AgNP content. The depth was estimated by the corrosion rate of the Ta_2_O_5_ standard.

# Antiviral assays


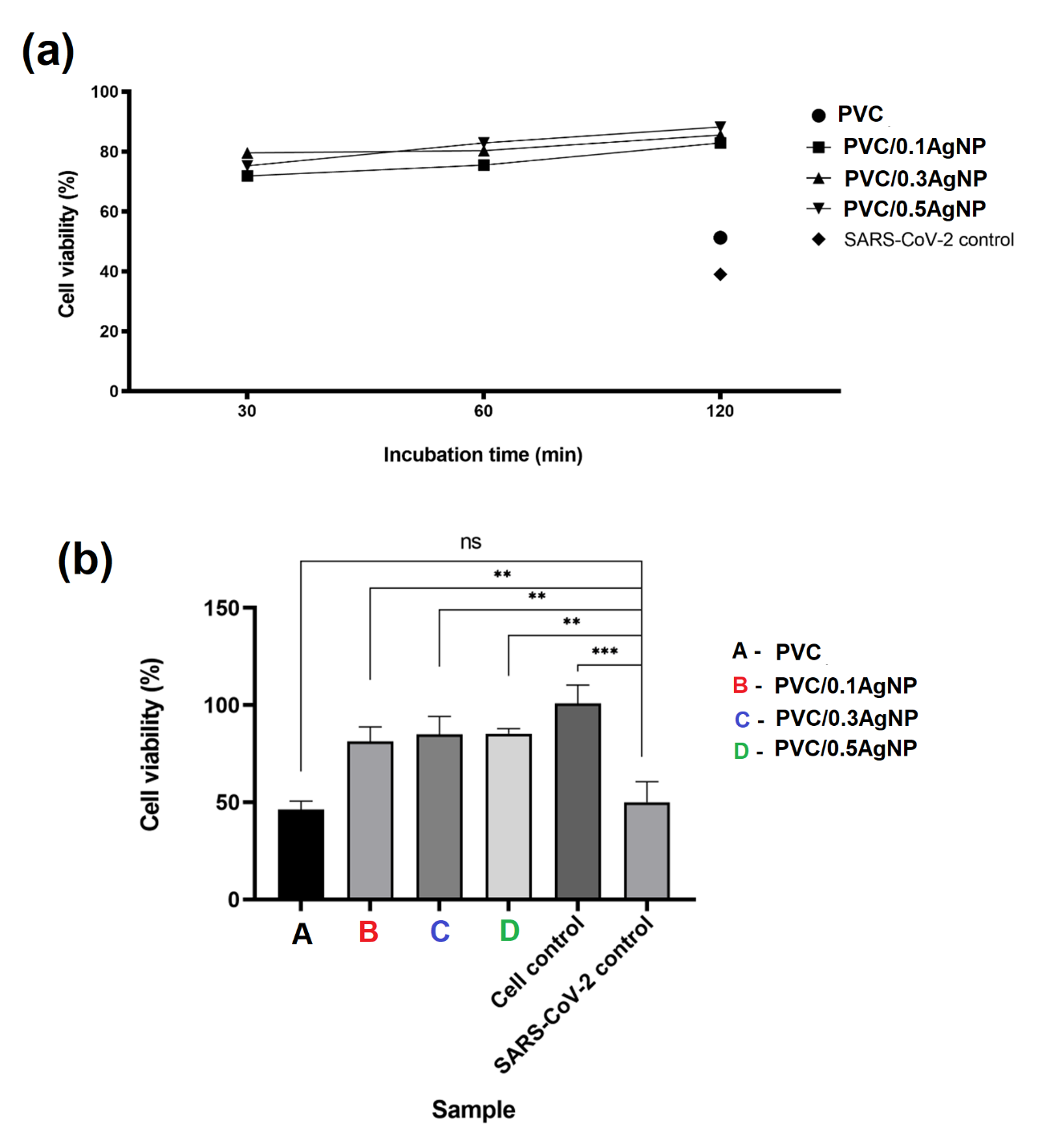


Figure 4S. (a) Cell viability within different direct contact times (incubation times) at the surfaces of the PVC and PVC/*X*AgNP nanocomposites: cell viability was measured by the MTT reduction assays using absorption measurements at 570 nm (SkanIt Software 2.4.5, Varioskan® Flash, Thermo Fisher, USA). (b) Cell viability at the incubation time of 120 min: bars represent average ± standard deviation (SD). ANOVA was applied (p=0.0001), followed by Dunn's variance test. Labels: ns= not significant; *= p<0.05; **= p<0.005; ***= p<0.005.
